# Thidiazuron Promotes Leaf Abscission by Regulating the Crosstalk Complexities between Ethylene, Auxin, and Cytokinin in Cotton

**DOI:** 10.3390/ijms23052696

**Published:** 2022-02-28

**Authors:** Fangjun Li, Qian Wu, Baopeng Liao, Keke Yu, Yini Huo, Lu Meng, Songman Wang, Baomin Wang, Mingwei Du, Xiaoli Tian, Zhaohu Li

**Affiliations:** 1 Engineering Research Center of Plant Growth Regulator, Ministry of Education/College of Agronomy and Biotechnology, China Agricultural University, Beijing 100193, China; lifangjun@cau.edu.cn (F.L.); 13617076259@163.com (B.L.); kekeyjsxz@163.com (K.Y.); huoyini819@163.com (Y.H.); ml513635063@163.com (L.M.); lovetochange@163.com (S.W.); wbaomin@263.net (B.W.); tianxl@cau.edu.cn (X.T.); lizhaohu@cau.edu.cn (Z.L.); 2 Institute of Agricultural Information, Jiangsu Academy of Agricultural Sciences, Nanjing 210014, China; wuqian@jaas.ac.cn; 3 High Latitude Crops Institute, Shanxi Agriculture University, Datong 037008, China

**Keywords:** thidiazuron (TDZ), leaf abscission, phytohormone, transcriptome

## Abstract

Thidiazuron (TDZ) is widely used as a defoliant to induce leaf abscission in cotton. However, the underlying molecular mechanism is still unclear. In this study, RNA-seq and enzyme-linked immunosorbent assays (ELISA) were performed to reveal the dynamic transcriptome profiling and the change of endogenous phytohormones upon TDZ treatment in leaf, petiole, and abscission zone (AZ). We found that TDZ induced the gene expression of ethylene biosynthesis and signal, and promoted ethylene accumulation earlier in leaf than that in AZ. While TDZ down-regulated indole-3-acetic acid (IAA) biosynthesis genes mainly in leaf and IAA signal and transport genes. Furthermore, the IAA content reduced more sharply in the leaf than that in AZ to change the auxin gradient for abscission. TDZ suppressed CTK biosynthesis genes and induced CTK metabolic genes to reduce the IPA accumulation for the reduction of ethylene sensitivity. Furthermore, TDZ regulated the gene expression of abscisic acid (ABA) biosynthesis and signal and induced ABA accumulation between 12–48 h, which could up-regulate ABA response factor genes and inhibit IAA transporter genes. Our data suggest that TDZ orchestrates metabolism and signal of ethylene, auxin, and cytokinin, and also the transport of auxin in leaf, petiole, and AZ, to control leaf abscission.

## 1. Introduction

Abscission, as an important biological process for plant survival and reproduction, is a highly coordinated event involving multiple changes in cell structure, metabolism, and gene expression [1]. Detachment of plant organs occurs at a predetermined position called the abscission zone (AZ) that develops at the junction between the leaving organ and the main plant body. When plants are subjected to abiotic/biological stresses or undergo senescence, the intercellular substance and cell wall of the separation layer cells degrade, leading to the organ finally detachment from the plant body [2]. The process of abscission has been proposed to comprise of four major steps including phase one, the cells differentiate into specialized cells namely abscission zone; phase two, acquisition of competence to respond to abscission signals; phase three, activation of abscission for organ shedding; phase four, sealing of the break by differentiation of a protective layer on the main body side of the AZ [3].

Early studies have provided many insights into the regulation mechanism of plant organ abscission. For phase one of the abscission process, Nonexpressor of Pathogenesis-Related (NPR) family genes *Blade On Petiole1* (*BOP1*) and *Blade On Petiole2* (*BOP2*) mediate the differentiation of the AZ in *Arabidopsis thaliana* [4]. In Arabidopsis, a MADS-box transcription factor *SEEDSTICK* (*STK*) controls cell division/differentiation to ensure the formation of the seed abscission zone [5]. The SLMBP21-J-MC complex, including JOINTLESS, MACROCALYX, and SLMBP21, three interacting proteins of the MADS-box family, is involved in the regulation of the development of the tomato flower abscission zone [6]. In Arabidopsis, the MADS-box domain proteins *AGAMOUS LIKE15* (*AGL15*) and *AGAMOUS LIKE18* (*AGL18*) participate in the shedding of flowers [7]. In phase two of the abscission process, a core signaling cascade, which is ethylene-independent, is uncovered to regulate abscission in Arabidopsis. Signaling peptide INFLORESCENCE DEFICIENT IN ABSCISSION (IDA) and two receptor-like kinases HAESA and HAESA-LIKE2 (HSL2) are comprised of the putative ligand–receptor system [8]. IDA binds and activates HAE/HSL2. Then, MITOGEN-ACTIVATED PROTEIN KINASE3/6 (MAPK3/6) is activated to inhibit the expression of the *BREVIPEDICELLUS* (*BP*)/*KNOTTED-LIKE FROM ARABIDOPSIS THALIANA1* (*KNAT1*), thus triggering the downstream *KNAT2/6* expression to promote the development of the abscission zones and initiate the shedding of the organs [8]. However, the IDA-like gene was up-regulated during abscission but the up-regulation was not limited to AZ, while none of the soybean HAE-like genes identified show a transcript expression pattern specific to the AZ, thus it is proposed that the IDA-HAE/HSL2 signal transduction pathway is not critical to the abscission process in tomato and soybean plants [9]. During phase three of the abscission process, xyloglucan endotransglucosylase hydrolase (XTH), polygalacturonase (PG), cellulase, pectinesterase, endoglucosidase, ribonuclease, expansin (EXP), and so on, participate in the degradation process of the abscission layer cells [10,11]. XTH is involved in the establishment of the cell wall [12]. Cellulase participates in the degradation of the cell wall by hydrolyzing cellulose. Pectinaseterase hydrolyzes the methyl ester in the pectin and promotes the production of pectic acid, which may be the substrate of PG. PG hydrolyzes the pectin substance in the intercellular layer during cell separation [13,14]. The effects of specific functional genes on organ shedding may be common to different species, but the signals that trigger their expression may be different [9]. Up to now, the molecular regulatory mechanism of sensing endogenous and exogenous signals and initiating the whole process of abscission is still unclear.

Plant hormones play a major role in regulating the abscission of plant organs. Particularly, ethylene regulates important abscission processes including cell wall modification, signal transduction, lipid transport, and lignin biosynthesis [15]. In Arabidopsis, ethylene-insensitive mutants *ETR1-1* and *EIN2* inhibit flower organ shedding [16]. In tomatoes, the suppression of *EIN3-LIKE* gene expression also inhibited the shedding of flower organs [17]. The regulation of abscission also involves auxin, which functions as a brake. High auxin concentrations in the AZ cells will inactivate organ abscission [18]. While, auxin response factors *ARF1*, *ARF2*, *ARF7*, and *ARF19* induce the floral organs shedding in Arabidopsis [19], which might be modulated by the changes in auxin gradients across AZs. In addition, auxin transporters delay leaf shedding via inhibition of polar auxin transport in abscission zones [18]. Auxin-ethylene crosstalk has been defined as one of the most important regulatory pathways in the control of organ abscission [15]. The crosstalk between cytokinin (CTK) and ethylene has also been shown to regulate the shedding of cotton leaves [15]. Other plant hormones, such as abscisic acid (ABA) and gibberellin (GA) indirectly induced by ethylene, also affect the formation of the abscission zone and the shedding of the organ [20,21]. Therefore, the abscission of plant organs seems to be affected by the balance and synergism of different plant hormones.

Cotton (*Gossypium hirsutum*) is one of the most economically valuable crops. In order to improve the efficiency of mechanical cotton harvesting, chemical defoliation is becoming an important agronomic practice. Chemical defoliation is a technique that uses plant growth regulators to regulate the growth and development of cotton, promotes endogenous ethylene synthesis and inhibit auxin transport, and finally induce early leaf abscission, boll dehiscence, and opening [22]. Thidiazuron (TDZ), a synthetic cytokinin-like molecule, as a defoliant is widely used to facilitate mechanical harvesting for many crops, especially cotton. The present researches mainly focus on its application technology, while the underlying molecular mechanism of TDZ on cotton leaf abscission is still unclear.

In this study, RNA-seq was performed to reveal the dynamical transcriptome change in cotton leaf, petiole, and AZ induced by TDZ. Plant hormone-related genes and transcription factors corresponding to the different stages of the abscission process were selected for further analysis. We demonstrate that TDZ mainly induces ethylene and ABA biosynthesis and signal while suppressing auxin and cytokinin biosynthesis, and also the transport of auxin from leaf to AZ, to control the cotton leaf abscission.

## 2. Results

### 2.1. Thidiazuron Induces Transcriptome Change in Leaf, Petiole, and AZ of Cotton

**Figure 1:**
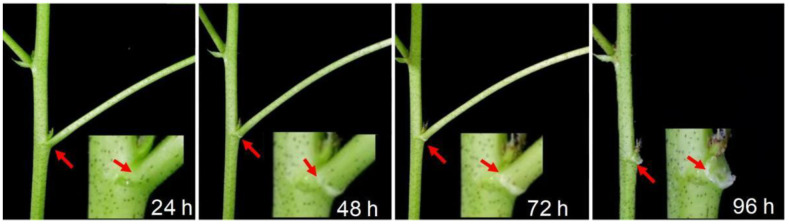
Effect of thidiazuron (TDZ) on cotton leaf abscission at 24, 48, 72, and 96 h after treatment; 750 mg/L TDZ was applied evenly to the whole plants at the six-leaf stage. The pictures were taken at 24, 48, 72, and 96 h, respectively, after treatment.

To study the effects of thidiazuron (TDZ) on cotton leaf abscission, we sprayed cotton leaves with TDZ and found that TDZ promoted the formation of abscission zone (AZ) at 48 h, and leaf abscission at 96 h (Figure 1).

**Figure 2:**
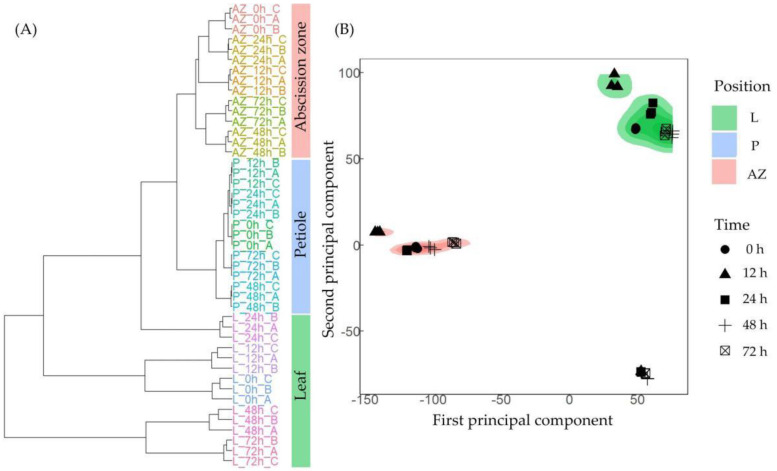
Transcriptome relationship among five time points of thidiazuron (TDZ) treatment in leaf (L), petiole (P), and abscission zone (AZ). (**A**) Cluster dendrogram showing three distinct tissues of leaf, petiole, and AZ. (**B**) Principal component analysis (PCA) of five time point samples in leaf, petiole, and AZ. Cotton leaves were treated with 750 mg/L thidiazuron (TDZ). Leaf, petiole, and AZ were sampled at 0, 12, 24, 48, and 72 h after treatment and were subjected to RNA-seq analyses.

**Figure 3:**
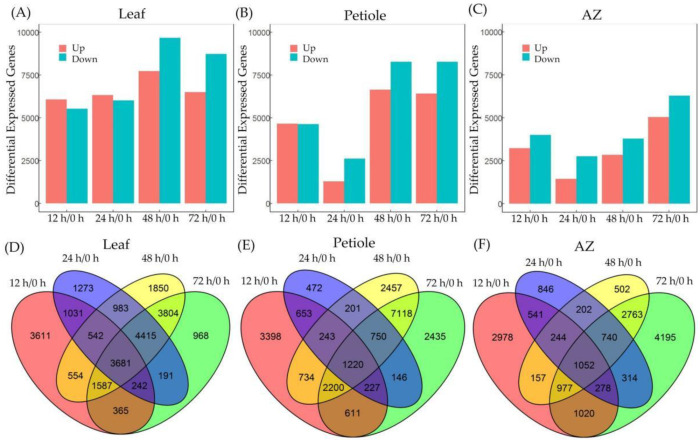
Differentially expressed genes (DEGs) regulated by TDZ. The total number of up- and down-regulated DEGs in leaf (**A**), petiole (**B**), and AZ (**C**) upon TDZ treatment. Venn diagram displaying DEGs and common genes in leaf (**D**), petiole (**E**), and AZ (**F**) upon TDZ treatment at four time points. DEGs were determined between 12 h and 0 h, 24 h and 0 h, 48 h and 0 h, 72 h and 0 h, respectively. The DEGs were controlled by FDR < 5% and |log2Fc| ≥ 1.

We further performed RNA-seq of leaf, petiole, and AZ to gain insight into the transcriptome dynamic of cotton leaf abscission induced by TDZ. The hierarchical clustering (Figure 2A) and principal component analysis (PCA) (Figure 2B) showed that these transcriptomes can be generally divided into three groups, with each group corresponding to a specific tissue and the three biological replicates of each time points have a strong correlation. Next, differentially expressed genes (DEGs) induced by TDZ were determined at all the detected time points in the leaf, petiole, and AZ, respectively. In leaf, 11,613, 12,358, 17,416, and 15,253 DEGs (Figure 3A), in petiole, 9286, 3912, 14,923, and 14,707 DEGs (Figure 3B), and in AZ, 7247, 4217, 6637, and 11,337 DEGs (Figure 3C), were found at the four indicated time points. The results showed that TDZ induced the most DEGs in leaf, while the least DEGs in AZ. Meanwhile, the most DEGs appeared at 48 h in leaf, 48–72 h in petiole, and 72 h in AZ. The results indirectly suggest that the effect of TDZ on the transcriptome change was gradually from leaf to petiole and finally to AZ.

There were 1052, 1220, and 3681 common DEGs in leaf (Figure 3D), petiole (Figure 3E), and AZ (Figure 3F), respectively, among all the time points. The expression dynamic of DEGs along the four time points was identified in three tissues by K-means clustering (Figure S1). The similarity of gene expression levels was analyzed according to Euclidean distance. Most of the DEGs showed continuously down-regulated or up-regulated; while some DEGs showed up-regulated first and then down-regulated or down-regulated first and then up-regulated. The dynamic expression of DEGs in leaf mostly changed from 24 h after TDZ treatment (Figure S1A), and that in petiole (Figure S1B) and AZ (Figure S1C) mostly changed from 48 h after TDZ treatment.

**Figure 4:**
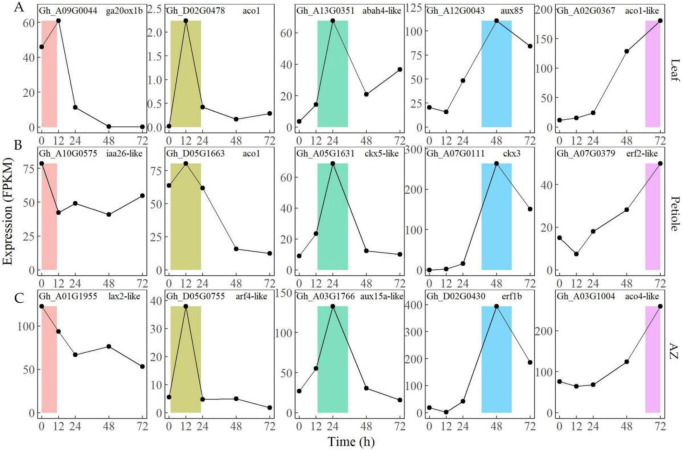
The expression (FPKM) of DEGs involved in plant hormone pathway upon TDZ treatment in leaf (**A**), petiole (**B**), and AZ (**C**) at indicated time points. The DEGs of 0, 12, 24, 48, and 72 h are shown in pink, yellow-green, green, blue, and purple, respectively.

To verify the DEGs’ response to TDZ treatment, several genes involved in the plant hormone pathway were selected to detect their expressing change by using FPKM in leaf (Figure 4A), petiole (Figure 4B), and AZ (Figure 4C) at indicated time points after TDZ treatment. Auxin transport gene *GhLAX2* (Gh_A01G1955), gibberellin biosynthesis gene *GhGA20ox1* (Gh_A09G0044), and auxin signaling gene *GhIAA26* (Gh_A10G0575) [23,24], were highly expressed before TDZ treatment in all the tissues. Auxin signaling gene *GhARF4* (Gh_D05G0755) and ethylene biosynthesis genes *GhACO1* (Gh_D02G0478) and *GhACO2* (Gh_D05G1663) [25], were highly expressed at 12 h after TDZ treatment in all the tissues. Auxin signaling gene *GhAUX15a* (Gh_A03G1766), abscisic acid metabolism gene *GhABAH4* (Gh_A13G0351), and cytokinin metabolism gene *GhCKX5* (Gh_A05G1631) [26], were highly expressed at 24 h after TDZ treatment in all the tissues. Ethylene signaling gene *GhERF1b* (Gh_D02G0430), auxin signaling gene *GhAUX85* (Gh_A12G0043), and cytokinin metabolism gene *GhCKX3* (Gh_A07G0111), were highly expressed at 48 h after TDZ treatment in all the tissues. Ethylene biosynthesis genes *GhACO4* (Gh_A03G1004) and *GhACO3* (Gh_A02G0367), and ethylene signaling gene *GhERF2* (Gh_A07G0379), were highly expressed at 72 h after TDZ treatment in all the tissues. Most of the gene expressing patterns selected here were consistent with previous reports [15,27]. To further validate the quality of the gene expression profiles, a total of 6 transcripts were randomly selected in AZ to compare the FPKM values and RT-qPCR data. The results showed that both of them largely corresponded to each other (cor > 0.58, average cor = 0.72) (Figure S2).

### 2.2. Thidiazuron Regulates the Expression of Plant Hormone Related Genes in Leaf, Petiole, and AZ

To identify the biological pathways involved in the abscission process upon TDZ treatment, we used KOBAS software to test the statistical enrichment of DEGs in KEGG pathways. The DEGs from leaf (Figure S3A), petiole (Figure S3B), and AZ (Figure S3C) were enriched in the plant hormone signal transduction pathway, carbon metabolism, starch and sucrose metabolism pathway, biosynthesis of amino acid, amino acid sugars, and nucleotide sugars metabolism, phenylpropanoid biosynthesis and so on. The most enriched DEGs were involved in the plant hormone signaling pathway.

**Figure 5:**
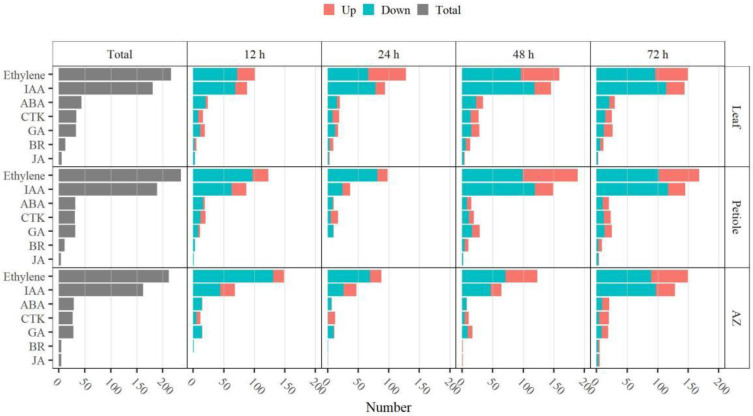
TDZ regulates the expression of phytohormone-related genes. The total number of up- and down-regulated DEGs related to plant hormones in leaf, petiole, and AZ at indicated time points after TDZ treatment were shown. DEGs were determined between 12 h and 0 h, 24 h and 0 h, 48 h and 0 h, 72 h and 0 h, respectively. The DEGs were controlled by FDR < 5% and |log2Fc| ≥ 1.

Phytohormones play a key role in the plant life cycle and are closely related to the organ abscission process [28]. In this study, we found 516, 522, and 459 DEGs were involved in hormone metabolism and signaling in leaf, petiole, and AZ upon TDZ treatment, respectively (Figure 5). Most of the DEGs were related to ethylene (216 DEGs in leaf, 235 DEGs in petiole, and 212 DEGs in AZ) and auxin (170 DEGs in leaf, 177 DEGs in petiole, and 153 DEGs in AZ). Meanwhile, a certain number of DEGs were related to ABA (44 DEGs in leaf, 32 DEGs in petiole, and 29 DEGs in AZ) and CTK (34 DEGs in leaf, 31 DEGs in petiole, and 27 DEGs in AZ); while others were related to GA (33 DEGs in leaf, 32 DEGs in petiole, and 28 DEGs in AZ), BR (13 DEGs in leaf, 11 DEGs in petiole, and 5 DEGs in AZ), and JA (6 DEGs in leaf, 4 DEGs in petiole, and 5 DEGs in AZ). Those DEGs were most enriched at 48 h in leaf, at 48–72 h in petiole, and at 72 h in AZ, suggesting that TDZ has earlier effects on phytohormone-related genes in leaf than that in petiole and AZ.

**Figure 6:**
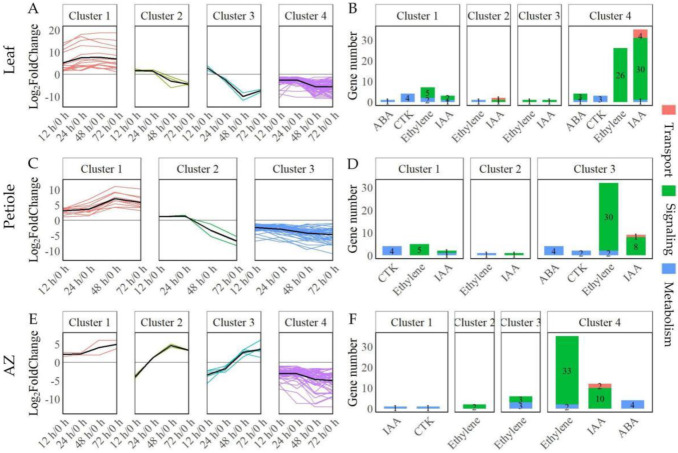
The dynamic expression and gene numbers of DEGs related to phytohormone in leaf, petiole, and AZ upon TDZ treatment. K-means clustering and gene numbers at four time points upon TDZ treatment in leaf (**A**,**B**), petiole (**C**,**D**), and AZ (**E**,**F**), respectively. DEGs were determined between 12 h and 0 h, 24 h and 0 h, 48 h and 0 h, 72 h and 0 h, respectively. The DEGs were controlled by FDR < 5% and |log2Fc| ≥ 1 at all four time points.

Next, we analyzed the dynamic expression patterns of DEGs related to plant hormones. In leaf, the dynamic expression patterns were divided into continuously up-regulated (Cluster 1), up-regulated at 12–24 h and down-regulated at 48–72 h (Cluster 2), up-regulated at 12 h and down-regulated at 24–72 h (Cluster 3), and continuously down-regulated (Cluster 4) (Figure 6A). Genes of ethylene metabolism were grouped into Cluster 1 and 2, and ethylene signaling were grouped into Cluster 1, 3, and 4; genes of IAA metabolism were grouped into Cluster 1 and 4, IAA signaling were grouped into Cluster 1–4, and IAA transport were grouped into Cluster 2 and 4; genes of ABA metabolism were grouped into Cluster1 and 4, ABA signaling were grouped into Cluster 4; genes of CTK metabolism were grouped into Cluster 1 and 4 (Figure 6B).

In petiole, the dynamic expression patterns were divided into continuously up-regulated (Cluster 1), up-regulated at 12–24 h and down-regulated at 48–72 h (Cluster 2), and continuously down-regulated (Cluster 3) (Figure 6C). Genes of ethylene metabolism were grouped into Cluster 2 and 3 and ethylene signaling were grouped into Cluster 1 and 3; genes of IAA metabolism were grouped into Cluster 1, IAA signal were grouped into Cluster 1, 2, and 3, and IAA transport were grouped into Cluster 3; genes of ABA metabolism were grouped into Cluster 3; genes of CTK metabolism were grouped into Cluster 1, 3 (Figure 6D). 

In AZ, the dynamic expression patterns were divided into continuously up-regulated (Cluster 1), down-regulated at 12 h and up-regulated at 24–72 h (Cluster 2), down-regulated at 12–24 h and up-regulated at 48–72 h (Cluster 3), and continuously down-regulated (Cluster 4) (Figure 6E). Genes of ethylene metabolism were in Cluster 3 and 4 and ethylene signaling were in Cluster 2–4; genes of IAA metabolism were grouped into Cluster 1 and IAA signaling and transport were grouped into Cluster 4; genes of CTK metabolism were grouped into Cluster 1; genes of ABA metabolism were grouped into Cluster 4 (Figure 6F).

### 2.3. Thidiazuron Regulates the Gene Expression of Ethylene Metabolism and Signaling

**Figure 7:**
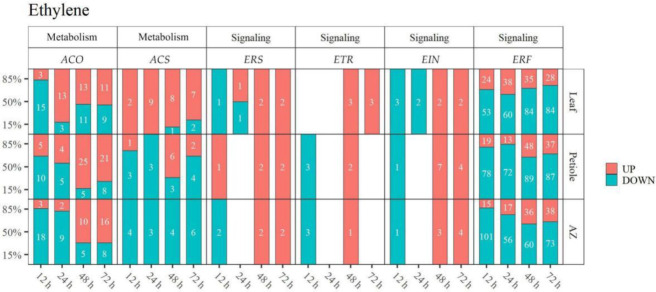
TDZ regulates the gene expression of ethylene metabolism and signal. DEGs were determined between 12 h and 0 h, 24 h and 0 h, 48 h and 0 h, 72 h and 0 h, respectively. The DEGs were controlled by FDR < 5% and |log2Fc| ≥ 1. White digits indicate the gene numbers.

Ethylene is a pivotal phytohormone involved in organ abscission [16]. We found that TDZ regulates the gene expression of ethylene metabolism and signaling (Figure 7, Supplementary Table S2). Ethylene synthesis genes *1-aminocyclopropane-1-carboxylic acid oxidase* (*ACOs*) and *1-aminocyclopropane-1-carboxylate synthase enzyme* (*ACSs*), and ethylene signaling genes *ERSs*, *ETRs*, *EINs*, and *ERFs* were differentially expressed upon TDZ treatment. Out of all the detected *ACO* genes, *ACO1-LIKE* and *ACO3-LIKE* were up-regulated from 24 h (37/60 = 61.7%) in leaf; and *ACO1* and *ACO3* were up-regulated from 48 h (46/59 = 80.0% in petiole and 26/39 = 66.7% in AZ) in petiole and AZ. Most of *ACS* genes (*ACS-LIKE*, *ACS7*, *ACS7-LIKE*) were up-regulated in leaf (26/29 = 89.7%), while down-regulated in AZ (17/17 = 100%). The ethylene receptor genes *ETRs* (*ETR2*) and *ERSs* (*ERS1*, *ERS1-LIKE*) were up-regulated from 48 in all the detected tissues. The ethylene signaling genes *EINs* (*EIN2-LIKE*, *EIN3*, *EIN3-LIKE*) were up-regulated from 48 h, and the ratio of up-regulated *ERFs* genes was increased from 24 h in leaf and from 48 h in petiole and AZ. Thus, it is likely that TDZ induces ethylene biosynthesis and signal earlier in the leaf than that in AZ.

### 2.4. Thidiazuron Regulates the Gene Expression of IAA Metabolism, Transport, and Signal

**Figure 8:**
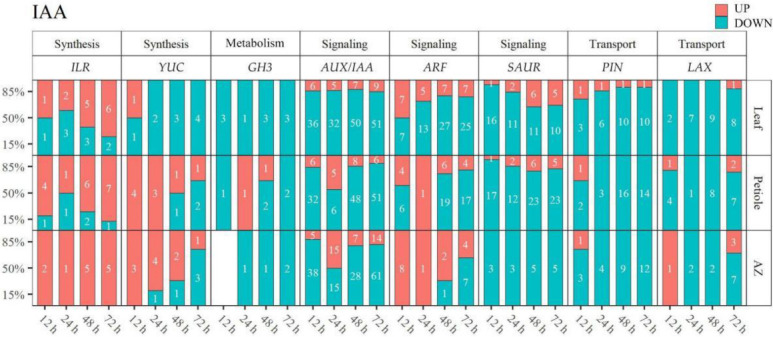
TDZ regulates the gene expression of IAA metabolism, transport, and signal. DEGs were determined between 12 h and 0 h, 24 h and 0 h, 48 h and 0 h, 72 h and 0 h, respectively. The DEGs were controlled by FDR < 5% and |log2Fc| ≥ 1. White digits indicate the gene numbers.

IAA-related DEGs were identified in leaf, petiole, and AZ, respectively (Figure 8, Supplementary Table S3). Auxin-responsive gene *GH3s* (*indole-3-acetic acid-amido synthetase*) that encode IAA-amide synthetase involved in the maintenance of hormonal homeostasis by conjugating free IAA to amino acids, were mainly down-regulated in leaf, petiole, and AZ. IAA-Leucine Resistant 1-like Hydrolase gene *ILRs* (*ILR1-LIKE3*, *ILR1-LIKE4, ILR1-LIKE6, ILR3-LIKE*), which encode IAA-amido hydrolase, were mainly up-regulated in petiole and AZ at indicated time points (31/35 = 88.6%). YUCCA gene *YUCs* (*YUCCA6-LIKE*, *YUCCA8, YUCCA10*), involving in catalyzing IAA biosynthesis, were mostly down-regulated from 12 h in leaf (10/11 = 90.9%), and from 72 h in petiole (2/3 = 66.7%) and AZ (3/4 = 75%). IAA signaling genes, *AUX/IAAs* (*AUX6B-LIKE*, *AUX15A-LIKE*, *AUX22, AUX22D-LIKE, AUX28-LIKE, AUXx15-LIKE, IAA1, IAA1-LIKE, IAA2-LIKE, IAA4-LIKE, IAA8-LIKE, IAA9, IAA9-LIKE, IAA11-LIKE, IAA13-LIKE,*, *IAA14-LIKE, IAA16-LIKE, IAA26-LIKE, IAA27, IAA27-LIKE, IAA29, ARG2-LIKE*, *ARG7-LIKE*, *ABP1*, *ABP19A-LIKE*, *ABPt85*, *AIR12-LIKE*) (447/540 = 82.8%) and *SAURs* (*SAUR32, SAUR32-LIKE, SAUR36-LIKE, SAUR41-LIKE, SAUR62-LIKE, SAUR71-LIKE, SAUR72, SAUR72-LIKE*) (139/167 = 83.2%), were mostly down-regulated; while most of the *ARFs* (*ARF2-LIKE, ARF3-LIKE, ARF4-LIKE, ARF5, ARF5-LIKE, ARF6-LIKE, ARF8-LIKE, ARF9-LIKE, ARF10-LIKE, ARF17, ARF18, ARF18-LIKE, ARF19-LIKE, ARF23-LIKE, AFB-LIKE*) were down-regulated in leaf (72/98 = 73.5%) and petiole (42/57 = 73.7%), and up-regulated in AZ (11/12 = 91.7%) at 12–48 h. Most of the *PINs* (*PIN1-LIKE, PIN3, PIN3-LIKE, PIN5, PIN6, PIN8, PIN1B, PIN1C*) (92/98 = 93.9%) and *LAXs* (*LAX2, LAX3, LAX5*) (57/65 = 87.7%), responsible for the transportation of IAA, were down-regulated in all the tissues at all the indicated time points. Together, we concluded that TDZ may reduce auxin biosynthesis first in leaf, then in AZ and petiole, and suppress IAA signal and transport in all the detected tissues.

### 2.5. Thidiazuron Regulates the Gene Expression Related to CTK and ABA

**Figure 9:**
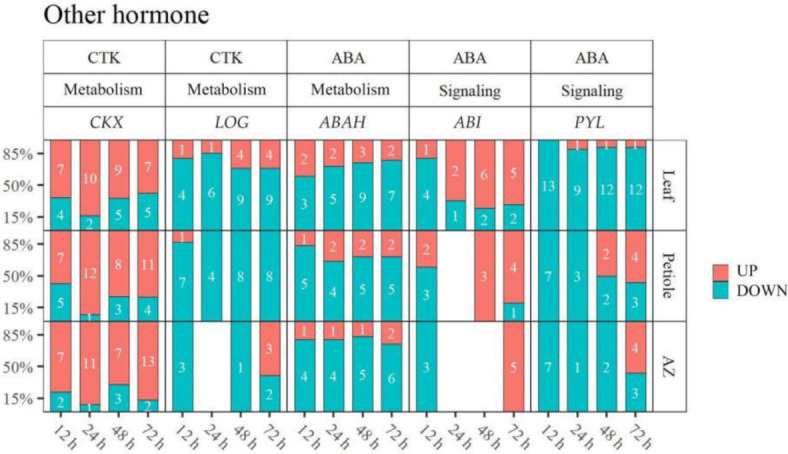
TDZ regulates the gene expression of metabolism and signal related to CTK and ABA. DEGs were determined between 12 h and 0 h, 24 h and 0 h, 48 h and 0 h, 72 h and 0 h, respectively. The DEGs were controlled by FDR < 5% and |log2Fc| ≥ 1. White digits indicate the gene numbers.

Meanwhile, CTK- and ABA-related genes were also regulated by TDZ (Figure 9, Supplementary Table S4). Cytokinin dehydrogenase gene *CKXs* (*CKX1-LIKE, CKX3, CKX3-LIKE, CKX5-LIKE, CKX6-LIKE, CKX7, CKX7-LIKE*), responsible for CTK metabolism, were mainly up-regulated (109/146 = 74.7%); and cytokinin activating enzyme gene *LOGs* (*LOG1-LIKE, LOG3, LOG5-LIKE, LOG8, LOG8-LIKE, LOG10*) were mainly down-regulated (61/72 = 84.2%) in all the detected tissues. ABA hydroxylase gene *ABAHs* (*ABAH1-LIKE, ABAH2-LIKE, ABAH3-LIKE, ABAH4, ABAH4-LIKE, ABAH1B, ABAH5-LIKE*) were mainly down-regulated (62/83 = 74.7%) in all the detected tissues. *ABIs* (*ABI1B, ABI5-LIKE*) as the regulator of ABA signal were mostly up-regulated (25/31 = 80.6%) from 24 h; while ABA receptor gene *PYLs* (*PYL2-LIKE, PYL4, PYL4-LIKE, PYL8-LIKE, PYL9-LIKE, PYR1-LIKE*) were mainly down-regulated (74/89 = 83.1%) after TDZ treatment.

### 2.6. Thidiazuron Modulates the Content of Multiple Plant Hormones

**Figure 10:**
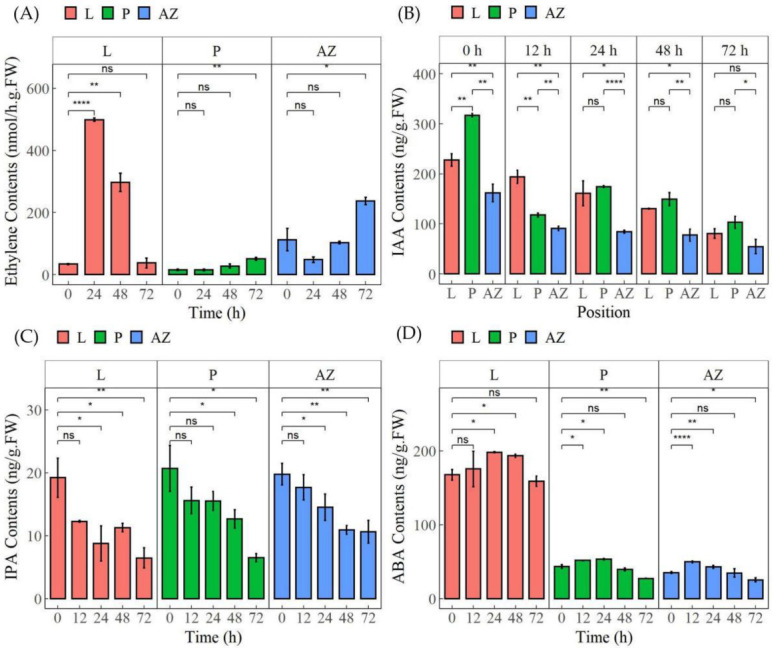
TDZ modulates the accumulation of ethylene (**A**), IAA (**B**), IPA (**C**), and ABA (**D**) in leaf, petiole, and AZ of cotton. Cotton leaves were treated with 750 mg/L TDZ. Leaf, petiole, and AZ were sampled at 0, 12, 24, 48, and 72 h after treatment and subjected to detect hormonal content. * *p* < 0.05, ** *p* < 0.01, **** *p* < 0.0001.

To further confirm the involvement of the above hormones, we detected the contents of ethylene, IAA, IPA (one of the cytokinins), and ABA in different tissues upon TDZ treatment. The data showed that TDZ induced the accumulation of ethylene in all the detected tissues (Figure 10A). In leaf, ethylene content was significantly induced 1354.5% at 24 h and 767.2% at 48 h; in petiole and AZ, it was gradually and significantly induced 232.9% and 110.6% at 72 h. Meanwhile, TDZ reduced the accumulation of IAA and IPA gradually in all the detected tissues (Figure 10B,C). IAA content both in leaf and petiole was significantly higher than that in AZ before TDZ treatment, while there were no differences between leaf and AZ after TDZ treatment for 72 h. IPA content was reduced 66.4% in leaf, 68.6% in petiole, and 46.2% in AZ after TDZ treatment for 72 h. Besides, TDZ induced ABA content at 12–48 h, then reduced at 72 h (Figure 10D). The data further confirmed that TDZ could orchestrate homeostasis of ethylene, auxin, cytokinin, and ABA in AZ, petiole, and leaf to regulate leaf abscission in cotton.

### 2.7. Thidiazuron Regulates the Gene Expression of Cell Cycle and Cell Wall in Abscission Zone

**Figure 11:**
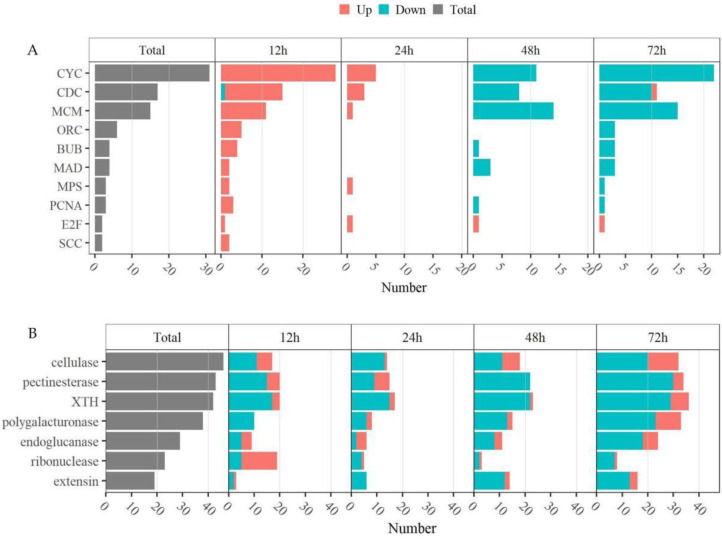
TDZ regulates the gene expression of the cell cycle (**A**) and cell wall (**B**) in AZ. DEGs were determined between 12 h and 0 h, 24 h and 0 h, 48 h and 0 h, 72 h and 0 h, respectively. The DEGs were controlled by FDR < 5% and |log2Fc| ≥ 1.

The first stage of the abscission process is the cell differentiation into abscission layer cells [3]. The genes related to the cell cycle play an important role in the regulation of cell differentiation. In plants, the cell cycle consists of four distinct phases (postmitotic interphase G1, DNA synthesis phase S, premitotic interphase G2, and mitosis/cytokinesis M) with two major check points (the G1/S check point and G2/M check point) [29], to ensure normal cell division. It is interesting that TDZ could induce quite a number of genes involved in the cell cycle. We found that cyclin gene *CYCs* (*CYC-A1-1-LIKE, CYC-A2-2-LIKE, CYC-A2-4-LIKE, CYC-B2-4-LIKE, CYC-B3-1, CYC-H1-1-LIKE, G2 mitotic-specific CYC C13-1-LIKE/CYC S13-7-LIKE/CYC-2-LIKE*; involved in the G1/S and G2/M transition phases), cell division cycle gene *CDCs* (*CDC2, CDC45, CDC6B-LIKE, CDC7, APC-LIKE*; involved in the mitosis phases), DNA replication permits *MCMs* (*MCM2, MCM3, MCM3-LIKE, MCM4, MCM5, MCM6, MCM7*) and *ORCs* (*ORC2, ORC4, ORC5, ORC6*) function in the S phases, mitotic checkpoint gene *BUBs* (*BUB1, BUBR1*), mitotic spindle checkpoint gene *MADs* (*MAD2*), and so on, were differentially expressed upon TDZ treatment, and most of them were up-regulated at 12–24 h and down-regulated at 48–72 h (Figure 11A, Supplementary Table S5), suggesting that TDZ could trigger the cell cycle and active cell differentiation to promote the first stage of leaf abscission process in cotton. 

The third stage of the abscission process is the degradation of the cell wall and intercellular matrix [3]. We found that genes of cellulase (*CESA1, CESA2, CESA3, CESA4, CESA5, CESA7, CSLE1, CSLD3, CSLD5, CSLE6, CSLG2, CSLG3, CSLH1*), pectinaseterase (*PAE5-LIKE, PAE6-LIKE, PAE9, PAE12-LIKE, PME3, PME3-LIKE, PME7, PME8, PME12, PME22, PME33, PME34, PME53, PME68, PME U1, PME PPE8B, QUA2*), XTH (*XTH2-LIKE, XTH8, XTH9, XTH10, XTH22-LIKE, XTH28, XTH30, XTH31-LIKE, XTH32, XTH33, XTH B*), polygalacturonase (*PGL3, QRT3*), endoglucanase (*EG1, EG2-LIKE, EG6, EG6-LIKE, EG8-LIKE, EG10-LIKE, EG11-LIKE, EG17, EG24-LIKE, EG CX-LIKE*), ribonuclease (*RNC, RTL2, MRNC, RNP3, RPP25L, RPP28, RNaseh2a*), and extension (*LRX1, LRX2, LRX4, LRX5, PEX1*) were differentially expressed upon TDZ treatment, and most of the above genes were down-regulated at all the detected time points; while there was a higher ratio of up-regulated genes from 72 h (Figure 11B, Supplementary Table S6). We speculated that the third stage of the abscission process had not been initiated at 72 h upon TDZ treatment.

### 2.8. Thidiazuron Regulates the Gene Expression of Transcript Factors in Abscission Zone

**Figure 12:**
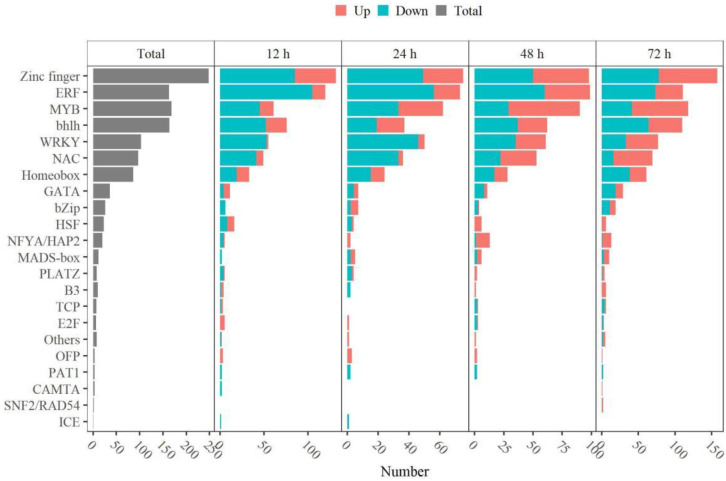
TDZ regulates the gene expression of transcript factors in AZ. DEGs were determined between 12 h and 0 h, 24 h and 0 h, 48 h and 0 h, 72 h and 0 h, respectively. The DEGs were controlled by FDR < 5% and |log2Fc| ≥ 1.

**Figure 13:**
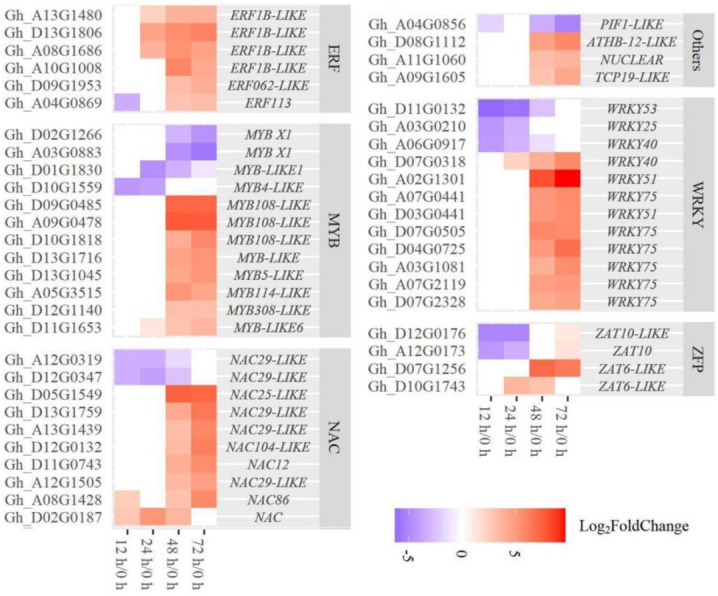
TDZ regulates the transcription factors genes in AZ. DEGs were determined between 12 h and 0 h, 24 h and 0 h, 48 h and 0 h, 72 h and 0 h, respectively. The DEGs were controlled by FDR < 5% and |log2Fc| ≥ 8 at least from two consecutive time points.

Transcription factors play an important role in regulating the expression of genes related to organ shedding [30,31,32,33]. In this study, 1197 differentially expressed transcription factors (TFs) were found in AZ after TDZ treatment, including *Zinc finger*, *MYB*, *bhlh*, *ERF*, *WRKY*, *NAC*, *Homeobox*, and so on (Figure 12, Supplementary Table S7). *ERF*, *bhlh*, and *Homeobox* TFs were mostly down-regulated at all four time points; while there were more *WRKY*, *Zinc finger*, *MYB,* and *NAC* TFs down-regulated at 12–24 h and more up-regulated genes at 48–72 h. The dynamic gene expression patterns of TFs were divided into four clusters including continuously up-regulated (Cluster 1), first up- and then down-regulated (Cluster 2), first down- and then up-regulated (Cluster 3), and continuously down-regulated (Cluster 4) (Figure S4A). More differentially expressed TFs were identified in Cluster 4 (Figure S4B). Among all the TFs, *WRKY75*, *WRKY51*, *WRKY40*, *MYB108*, *MYB114*, *MYB308*, *MYB5*, *NAC12*, *NAC25*, *NAC29*, *NAC86*, *NAC104*, *ZAT6*, *ERF62*, and *ERF1B* were up-regulated; while *WRKY53*, *WRKY40*, *WRKY25*, *MYB4*, *NAC29*, and *ZAT10* were down-regulated in AZ (Figure 13).

## 3. Discussion

Thidiazuron (TDZ), a cytokinin synthetic analog, is a defoliant widely used in cotton production to enhance the efficiency of mechanical harvesting of cotton [22]. It has been reported that TDZ reduces basipetal auxin transport in petiole segments and enhances endogenous ethylene evolution during leaf abscission [34]. With RNA-Seq analysis, a large number of DEGs induced by TDZ, especially genes related to hormone metabolism and signaling, were determined in leaf, petiole, and AZ of cotton. The results showed that most of DEGs were related to ethylene and IAA, and a certain number of DEGs were related to ABA and CTK upon TDZ treatment (Figure 5 and Figure S3), suggesting that TDZ promotes the cotton leaf shedding through regulating a complex regulatory network of ethylene, IAA, CTK, and ABA.

### 3.1. Thidiazuron Promotes Cotton Leaf Abscission by Activating Gene Expression of Ethylene Synthesis and Signaling

Plants produce a lot of ethylene before organ shedding, which plays an important role in organ separation and abscission [1]. Ethylene biosynthesis starts with the conversion of Sadenosyl-L-methione (SAM) into 1-aminocyclopropane-1-carboxylic acid (ACC) catalyzed by the enzyme 1-aminocyclopropane-1-carboxylate synthase (ACS). Then, ACC can be converted into either 1-(malonylamino) cyclopropane-1-carboxylic acid (MACC) by ACC N-malonyl transferase, or to the end product ethylene by 1-aminocyclopropane-1-carboxylate oxidase (ACO). Here, the results showed that the transcripts of *ACO*s were mostly up-regulated from 24 h in leaf, and from 48 h in petiole and AZ upon TDZ treatment (Figure 7). Consistently, the pick of ethylene content appeared much earlier in the leaf than that in petiole and AZ (Figure 10). The data indicated that TDZ could induce ethylene biosynthesis and accumulation in the leaf, which may further promote ethylene accumulation in AZ.

It has been reported that blocking ethylene signals can cause abnormal organ shedding. For example, ethylene-insensitive mutants *ETR1-1* and *EIN2* inhibit floral organ shedding in Arabidopsis [16]. Inhibition of *EIN3-LIKE* gene expression inhibits flower abscission and fruit ripening in tomatoes [17]. Our results showed that *ETR*, *ERS*, and *EIN* genes were up-regulated at 48–72 h after TDZ treatment (Figure 7). It is probably that TDZ first induces ethylene receptor genes (*ETR1*, *ETR2*, *ERS1*, and *ERS2*) to perceive ethylene signal and activate the positive regulatory factors *EIN2* and *EIN3*, promoting the expression of secondary transcription factor *ERFs* (*ERF1, ERF2, ERF3, ERF4, ERF5, ERF12, ERF13, ERF14, ERF34, ERF54, ERF61, ERF62, ERF96, ERF98, ERF107, ERF113, ERF114, ABR1, CRF1, CRF4, CRF5, RAP2, TINY, ANT, BBM*), which finally promotes cotton leaf abscission.

### 3.2. Thidiazuron Promotes Cotton Leaf Abscission by Regulating the Gene Expression of Auxin Synthesis, Transport, and Signaling

According to the theory of “Auxin gradient” in the process of plant abscission, the concentration gradient of auxin across the petiole and the two sides of the AZ is the decisive factor of plant abscission [18]. When the auxin content near the distal end of AZ is higher than that near the axial end, the organs do not shed; when the auxin content is almost equal on both sides of AZ, abscission occurs; when auxin content is high near the axial end, the abscission is accelerated. Our results showed that TDZ down-regulated IAA synthesis gene *YUCs* (*YUCCA6-LIKE, YUCCA10*) from 24 h in leaf and from 72 h in petiole and AZ, suggesting that the auxin content in petiole and AZ may reduce more slowly than that in leaf (Figure 8). Consistently, auxin content in leaf and petiole showed sharper reduction than that in AZ (Figure 10B), indicating that TDZ could change the appropriate auxin gradient to facilitate leaf abscission.

If the flux of IAA to the abscission zone region is maintained, cell separation is inhibited and abscission does not occur [35]. Treatment with inhibitors of auxin polar transport could regulate IAA metabolism, transport, and signal genes expression to promote organs abscission [18]. Here we showed that the IAA transporters genes *PINs* and *LAXs* were mostly down-regulated in all the detected tissues after TDZ treatment (Figure 8), indicating that TDZ could also promote leaf abscission via the inhibition of auxin polar transport [34].

Auxin response factors *ARF1*, *ARF2*, *ARF7*, and *ARF19* induce leaf senescence and floral organ abscission in Arabidopsis [19]. Consistently, we showed that most of *ARFs*, such as *ARF4-LIKE*, *ARF5-LIKE*, *ARF10-LIKE*, *ARF23-LIKE*, were up-regulated at 12–48 h in the AZ after TDZ treatment.

### 3.3. Thidiazuron Regulates Genes Expression of CTK Synthesis as Well as ABA Synthesis and Signaling

CTK plays an important role in regulating plant development, cell differentiation, and cell division. A high concentration of CTK inhibits the formation of abscission layers [1], but some compounds that synthesize CTK promote the formation of abscission layers mediated by ethylene and are used in production as a defoliant [36]. TDZ used in this study was a defoliant with cytokinin activity. We showed that cytokinin dehydrogenase gene *CKX*s (*CKX1-LIKE, CKX3, CKX3-LIKE, CKX5-LIKE, CKX6-LIKE, CKX7, CKX7-LIKE*) were mostly up-regulated, and cytokinin-activated enzyme gene *LOG*s were mostly down-regulated after TDZ treatment (Figure 9). Consistently, the content of CTK was reduced in all the detected tissues. The data indicate that TDZ induced leaf abscission is caused by the reduction of CTK accumulation.

ABA, an endogenous hormone causing abscission, is increased in senescent leaves and some abscission fruits. The effect of ABA on organ abscission is indirectly realized by inhibiting auxin conduction or stimulating ethylene production [37]. Our results showed that ABA metabolism genes (*ABAH1-LIKE, ABAH2-LIKE, ABAH3-LIKE, ABAH4, ABAH4-LIKE, ABAH1B, ABAH5-LIKE*) were down-regulated after TDZ treatment; and ABA signaling genes *ABIs* (*ABI1B, ABI5-LIKE*) and *PYL*s (*PYL2-LIKE, PYL4, PYL4-LIKE, PYL8-LIKE, PYL9-LIKE, PYR1-LIKE*) were activated (Figure 9). Among them *ABI4* may further inhibit the expression of IAA transporter gene *PIN1* [38], and ABA receptor gene *PYL8* may directly regulate *MYB77* transcription factor to up-regulate the expression of IAA responsive gene ARFs [39]. Thus, indirectly promoting leaf abscission.

In conclusion, TDZ may indirectly promote cotton leaf abscission by suppressing the synthesis of CTK genes and inducing ABA synthesis and signal genes, to further stimulate ethylene production and inhibit IAA transport.

### 3.4. The Balance and Synergism of Ethylene, IAA, and Cytokinin Regulates the Cotton Leaf Abscission

With the development of the studies on organ abscission, it is generally considered that the balance between auxin and ethylene is the key factor affecting the separation of abscission layers [1,15,16]. The activation of abscission begins with the reduction of auxin transport or signal in the abscission layer and then is initiated by ethylene. Auxin can inactivate the cells of AZ and make them insensitive to ethylene signals, thus inhibiting shedding [16]. Here, we showed that TDZ reduced IAA content and transport, while induced ethylene content and signal genes in all the detected tissues (Figure 7, Figure 8 and Figure 10). Furthermore, several *ILRs* gene encoding the IAA-amino acid conjugate hydrolase were found to be up-regulated after TDZ treatment, which may increase the AZ sensitivity to ethylene and promote abscission [40]. Meanwhile, the increase of endogenous ethylene and the degradation of endogenous CTK can lead to the degradation of the cell wall and the separation of cells [15]. Ethylene could induce the expression of CKX, leading to CTK degradation; while inhibiting GhCKX3 impedes ethylene synthesis and signaling, thus reducing ethylene sensitivity [15]. Our data showed that TDZ inhibited CTK synthesis genes, while induced CTK metabolism genes, thus leading to the activation of ethylene biosynthesis genes (Figure 7, Figure 8 and Figure 9). Together, we speculate that the balance and synergism in metabolism and signal transduction of IAA, CTK, and ethylene play important roles in TDZ mediated cotton leaf abscission. However, the specific interaction mechanism of ethylene, CTK, and IAA needs to be further explored.

### 3.5. Differential Expression of Transcription Factors in Different Stages of Abscission Process

The effects of ethylene and auxin on plant development are regulated by several transcription factors, including ethylene transcription factor *ERF*, auxin response factor *ARF*, *NAC, MYB* transcription factors, and so on. Our results showed that TDZ could regulate the expression of quite a mount transcription factors, including *Zinc finger*, *MYB*, *bhlh*, *ERF*, *WRKY*, *NAC*, *Homeobox,* and so on (Figure 12), and some transcription factors have been reported involved in plant organ abscission. Over-expressed MADS-box *AGL15* delay floral organs shedding [7]. The MADS-box *MCM1* has an important regulatory function at two points M/G1 and G2/M in the cell cycle [41]. Here, TDZ induced the *MCM1*, which may regulate M/G1 and G2/M to trigger the cell cycle. Zinc finger transcription factors affect the transition to the DNA synthesis phase of the cell cycle [42]. In *Arabidopsis thaliana*, over-expression of Zinc finger protein (*AtZFP2*) delays the formation of floral abscission layers and the shedding of floral organs [43]. In addition, *ZFP8* and *ZFP6*, as cytokinin inducers, regulate cell differentiation at different stages of plant development [44,45]. We showed that the expression of *ZAT10* was down-regulated at 12–24 h; while the expression of *ZAT6* was up-regulated at 24–72 h (Figure 13), indicating that TDZ may function in the process of abscission layer differentiation through regulation of the S phase in the cell cycle.

WRKY transcription factor family genes, such as *WRKY6*, *WRKY22*, *WRKY42*, *WRKY45*, *WRKY53*, *WRKY54*, *WRKY57, WRKY70, WRKY71, WRKY75,* and *WRKY91* in Arabidopsis, have a mutual regulation relationship and play a positive or negative role in leaf senescence [31,32,46,47,48]. *WRKY6* and *WRKY75* play a positive role in leaf senescence. *WRKY75* is a direct target gene for *EIN3*, and the WRKY75-SA-ROS amplification loop is one of the factors related to ethylene-induced leaf senescence [46]. In this study, five *WRKY75*, two *WRKY51,* and one *WRKY40* transcription factors were up-regulated at 48–72 h after TDZ treatment (Figure 13), which may be involved in ethylene signal transduction to regulate the abscission process of cotton leaf.

In cassava, the MYB transcription factor family gene plays an important role in regulating the separation of abscission layers [49]. The expression of *MeMYB15* and *MeMYB73* is higher in the early and middle stages of leaf abscission; and seven R2R3 MYB genes are over-expressed in the late stages of leaf abscission, including one *MeMYB78*, one *MeMYB3*, two *MeMYB4*, and three *MeMYB62* genes. TDZ could induce the expression of three *MYB108*, one *MYB114*, *MYB5,* and *MYB308* at 48–72 h (Figure 13). Furthermore, it has been reported that *MdNAC047* regulates the expression of the ethylene response gene in apples under salt stress [30]. Here, we found that three *NAC29*, one *NAC12*, *NAC25*, *NAC86,* and *NAC104* were up-regulated upon TDZ treatment (Figure 13).

In the field, TDZ-induced cotton leaf defoliation is often affected by shading, drought, nitrogen supply, and so on. How to defoliate efficiently is still an important question. Our results provide new insight into further understanding the physiological and molecular mechanisms of TDZ-induced cotton leaf defoliation and are also helpful for dealing with the environmental influences on defoliation. In this study, a number of candidate genes, such as *ACO1/3*, *YUCCA6/10*, and *ILR1* in ethylene and IAA synthesis, *PIN5/6/8* in IAA transporter, *CKX3/5/7* in cytokinin metabolism, as well as the transcription factor genes *NAC12/25/29/104*, *WRKY40/51/75*, *MYB5/108/114/308*, and *ERF1B*, were identified as key players to control the abscission, which can potentially be exploited to regulate cotton defoliation via gene overexpression driven by the AZ-specific promoter or gene editing.

## 4. Materials and Methods

### 4.1. Plant Materials and Growth Conditions

Cotton (*Gossypium hirsutum Linn*., Xinshi17) seeds were provided by Hebei Guoxin Rural Technical Service Association. Seeds were surface-sterilized by soaking in 9% H_2_O_2_ for 30 min, then rinsed with tap water and soaked in deionized water for 36 h. Seeds of uniform size were selected and germinated in a silver sand bed for four days. Then, cotton seedlings were cultured in Hoagland nutrient solution, containing 2.5 mM Ca(NO_3_)_2_, 1 mM MgSO_4_, 0.5 mM (NH_4_)H_2_PO_4_, 2 × 10^−4^ mM CuSO_4_, 1 × 10^−3^ mM ZnSO_4_, 0.1 mM FeNaEDTA, 2 × 10^−2^ mM H_3_BO_3_, 5 × 10^−6^ mM (NH_4_)_6_Mo_7_O_24_, 1 × 10^−3^ mM MnSO_4_, and 0.1 mM K_2_SO_4_. The experiment was carried out in a customized growth room at China Agricultural University and the growth room was subject to controlled conditions with 14 h light/10 h dark at 24 ± 0.5/20 ± 0.5 °C, 70–80% relative humidity, and 600 ± 30 µmol m^−2^ s^−1^ photosynthetically active radiation.

### 4.2. Experimental Design

Thidiazuron (TDZ) (Sichuan Guoguang Agrochemical Co., Ltd., Sichuan, China) of 750 mg/L was applied evenly to the whole plants at the six-leaf stage. The pictures of leaf abscission were taken at 24 h, 48 h, 72 h, and 96 h, respectively. For RNA-seq and determination of plant hormone, TDZ was applied evenly to the fourth leaf of cotton seedlings. Then, leaf (without the leaf vein), petiole (5–6 mm long in the middle of the petiole), and abscission zones (AZ, 3–4 mm from petiole to distal end) were carefully sampled at 0 h, 12 h, 24 h, 48 h, 72 h, respectively, and stored at −80 °C. Three independent biological replicates were performed for each treatment. Each replicate included 10 individual cotton seedlings.

### 4.3. RNA Extraction, cDNA Library Preparation and Sequencing for RNA-Seq

Total RNA was extracted with the RNAprep Pure Plant Kit (Tsingke, TSP412, Beijing, China). Before generating the sequencing libraries, RNA purity was checked using the NanoPhotometer spectrophotometer (IMPLEN, CA, USA). Sequencing libraries were generated using NEBNext UltraTM RNA Library Prep Kit for Illumina (NEB, Ipswich, MA, USA) following the manufacturer’s recommendations and index codes were added to attribute sequences to each sample. RNA integrity and quality were assessed by the Agilent RNA 6000 Nano Chip in the Agilent 2100 Bioanalyzer (Agilent Technologies, Santa Clara, CA, USA). Then, 2 µg total RNA per sample was used as input material for the mRNA sample preparations.

The clustering of the index-coded samples was performed on a cBot Cluster Generation System using TruSeq PE Cluster Kit v4-cBot-HS (Illumina) according to the manufacturer’s instructions. After cluster generation, the library preparations were sequenced on an Illumina Hiseq 4000 platform and paired-end 150bp reads were generated.

### 4.4. Statistical Analysis of RNA-Seq

Raw data (raw reads) of fastq format were firstly processed through in-house Perl scripts. In this step, clean data (clean reads) were obtained by removing reads containing adapter and ploy-N, and low-quality reads from raw data. At the same time, Q20, Q30, GC-content, and sequence duplication levels of the clean data were calculated. All the downstream analyses were based on clean data with high quality. The Gossypium hirsutum genome database was used as a reference sequence. Clean reads were mapped to the reference genome using TopHat2.

Gene expression was normalized as FPKM (fragments per kilobase of exon model per million mapped reads) values. HTSeq v0.5.3 (EMBL, Heidelberg, Germany) was used to count the reads numbers mapped to each gene. FPKM >1 was used as the threshold to determine whether the gene was expressed in subsequent analysis. Sample relationships were analyzed by a Principal component analysis (PCA). PCA was conducted by using the prcomp function in R with default settings.

### 4.5. Quantitative Reverse Transcription PCR (RT-qPCR) Analysis

cDNA synthesis was performed with Superscript II reverse transcriptase (Invitrogen, Carabas, California, USA) according to the manufacturer’s instructions. qRT-PCR was performed using SYBR Green Master Mix and conducted in an Applied Biosystems 7500 Fast Real-Time PCR System (Applied Biosystems, Waltham, CA, USA). The expression level of each gene was determined relative to ACTIN9 as a housekeeping gene and was calculated using the 2^−ΔΔCT^ method [50]. The primers used in this study were listed in Supplementary Table S1.

### 4.6. Analysis of Differential Gene Expression and KEGG Enrichment

Differential expression analysis of each two replicates was performed using the DESeq R package. DESeq provides statistical routines for determining differential expression in digital gene expression data using a model based on the negative binomial distribution. The resulting *P* values were adjusted using Benjamini and Hochberg’s approach for controlling the false discovery rate. Genes with an adjusted *P*-value < 0.05 were assigned as differentially expressed. Euclidean algorithm-based K-means clustering was performed to generate the expression clusters of gene expression dynamics along four time points. For understanding high-level functions and utilities of the biological system, such as the cell, the organism, and the ecosystem at the molecular level, we used KOBAS software to test the statistical enrichment of DEGs in KEGG pathways (http://www.genome.jp/kegg/, accessed on 1 January 2022).

### 4.7. Measurement of Endogenous Hormonal Contents

Plant hormones, ethylene, IAA, IPA, and ABA in leaf, petiole, and AZ were extracted and purified according to the protocol described in Yang et al. [51]. Briefly, the samples of leaf, petiole, and abscission zone were harvested and washed with de-ionized water; then samples of 0.5 g were homogenized in 2 mL 80% methanol and stored at −20 °C for 48 h. The extract was centrifuged at 4000 g for 15 min at 4 °C, and then the supernatant was passed through C18 Sep-Pak cartridges (Waters Corp., Millford, MA, USA). The pellets were re-suspended with 10 mL of 100% (*v*/*v*) methanol and 10 mL of ether. Afterward, the eluate was dried down by pure N2 at 20 °C, and then stored at −40 °C. The concentration of plant hormones was determined by the ELISA technique following the protocol described in Zhao et al. [52]. For ethylene production measurement, the samples of leaf, petiole, and abscission zone were excised from cotton plants and put into a 10 mL airtight glass container with a rubber cap immediately. After 2 h standing under room conditions, the 2 mL gas sample was determined using a gas chromatograph (GC-2010, Shimadzu, Kyoto, Japan) equipped with a flame ionization detector and an activated alumina column according to Xue et al. [53].

## 5. Conclusions

**Figure 14:**
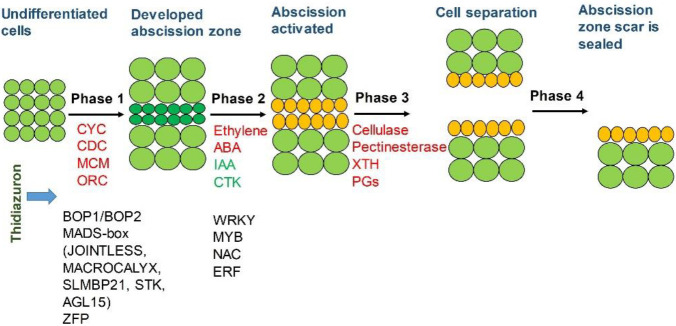
DEGs involved in the regulation of cotton leaf abscission at four abscission stages. In phase one, transcription factor genes of BOP1/BOP2 family, MADS family (JOINTLESS, MACROCALYX, SlMBP21, and STK), and zinc finger family were regulated by TDZ to promote the abscission differentiation. Meanwhile, CYC, CDC, MCM, and ORC were induced to promote the cell cycle. In phase two, TDZ promotes the biosynthesis gene expression and accumulation of ethylene and ABA, while suppressing the biosynthesis gene expression and accumulation of IAA and CTK. Meanwhile, TDZ regulates the gene expression of the transcription factors, including WRKY, MYB, NAC, and ERF regulate ethylene and IAA to promote the separation of AZ. In phase three, TDZ induces the gene expression related to cellulase, pectinesterase, XTH, and PGs to activate the degradation of the cell wall and intercellular substance for leaf shed off.

Thidiazuron-regulated genes’ function in the four stages of abscission was summarized (Figure 14). In phase one, TDZ induced the cell cyclin-related genes *CYCs*, *CDCs*, *MCMs*, and *ORCs* and regulates the involved transcription factor genes of BOP1/BOP2, MADS-box, and ZFP to promote the cell cycle. In phase two, the synthesis genes of ethylene and ABA were promoted, while the synthesis genes of IAA and CTK were suppressed. Consistently, TDZ induced the accumulation of ethylene and ABA, but reduced the accumulation of IAA and IPA gradually in all the detected tissues, to induce the abscission signal. Meanwhile, IAA transporter genes *PINs* were continuously down-regulated, which may change the auxin gradient in the AZ to benefit organ abscission. Furthermore, the transcription factors regulated by ethylene and IAA signal, including *WRKYs*, *MYBs*, *NACs,* and *ERFs*, were modulated to co-regulate the separation of AZ. In phase three, a small number of genes related to cellulase, pectinesterase, XTH, and PGs were being induced to initiate the degradation of the cell wall and intercellular substance. Together, TDZ initiates leaf abscission by the activation of the cell cycle to trigger cell differentiation into the AZ cells, then mainly induces ethylene and ABA biosynthesis and signal, while restrains auxin and cytokinin biosynthesis, and the transport of auxin from leaf to AZ, to promote leaf abscission.

## Data Availability

The datasets supporting the conclusions of this article are available in https://github.com/wuqiangithub/TDZ-RNA-SEQ-ABSCISSION, accessed on 24 January 2022.

## Supplementary Materials

The following supporting information can be downloaded at: https://www.mdpi.com/article/10.3390/ijms23052696/s1.

## References

[1] Gulfishan M., Jahan A., Bhat T.A., Sahab D., Sarwat M., Tuteja N. (2019). Chapter 16—Plant Senescence and Organ Abscission. Senescence Signalling and Control in Plants.

[2] Patharkar O.R., Walker J.C. (2019). Connections between Abscission, Dehiscence, Pathogen Defense, Drought Tolerance, and Senescence. Plant Sci..

[3] Patharkar O.R., Walker J.C. (2018). Advances in Abscission Signaling. J. Exp. Bot..

[4] McKim S.M., Stenvik G.E., Butenko M.A., Kristiansen W., Cho S.K., Hepworth S.R., Aalen R.B., Haughn G.W. (2008). The BLADE-ON-PETIOLE Genes Are Essential for Abscission Zone Formation in Arabidopsis. Development.

[5] Pinyopich A., Ditta G.S., Savidge B., Liljegren S.J., Baumann E., Wisman E., Yanofsky M.F. (2003). Assessing the Redundancy of MADS-Box Genes during Carpel and Ovule Development. Nature.

[6] Liu D., Wang D., Qin Z., Zhang D., Yin L., Wu L., Colasanti J., Li A., Mao L. (2014). The SEPALLATA MADS-Box Protein SLMBP21 Forms Protein Complexes with JOINTLESS and MACROCALYX as a Transcription Activator for Development of the Tomato Flower Abscission Zone. Plant J..

[7] Adamczyk B.J., Lehti-Shiu M.D., Fernandez D.E. (2007). The MADS Domain Factors AGL15 and AGL18 Act Redundantly as Repressors of the Floral Transition in Arabidopsis. Plant J..

[8] Shi C.L., Stenvik G.E., Vie A.K., Bones A.M., Pautot V., Proveniers M., Aalen R.B., Butenko M.A. (2011). Arabidopsis Class I KNOTTED-Like Homeobox Proteins Act Downstream in the IDA-HAE/HSL2 Floral Abscission Signaling Pathway. Plant Cell.

[9] Tucker M.L., Yang R. (2012). IDA-like Gene Expression in Soybean and Tomato Leaf Abscission and Requirement for a Diffusible Stelar Abscission Signal. AoB Plants.

[10] Kim J., Sundaresan S., Philosoph-Hadas S., Yang R., Meir S., Tucker M.L. (2015). Examination of the Abscission-Associated Transcriptomes for Soybean, Tomato, and Arabidopsis Highlights the Conserved Biosynthesis of an Extensible Extracellular Matrix and Boundary Layer. Front. Plant Sci..

[11] Kim J., Chun J.P., Tucker M.L. (2019). Transcriptional Regulation of Abscission Zones. Plants.

[12] Shinohara N., Nishitani K. (2021). Cryogenian Origin and Subsequent Diversification of the Plant Cell-Wall Enzyme XTH Family. Plant Cell Physiol..

[13] Yang Y., Anderson C.T., Cao J. (2021). Polygalacturonase45 Cleaves Pectin and Links Cell Proliferation and Morphogenesis to Leaf Curvature in Arabidopsis Thaliana. Plant J..

[14] Zhai Z., Feng C., Wang Y., Sun Y., Peng X., Xiao Y., Zhang X., Zhou X., Jiao J., Wang W. (2021). Genome-Wide Identification of the Xyloglucan Endotransglucosylase/Hydrolase (XTH) and Polygalacturonase (PG) Genes and Characterization of Their Role in Fruit Softening of Sweet Cherry. Int. J. Mol. Sci..

[15] Xu J., Chen L., Sun H., Wusiman N., Sun W., Li B., Gao Y., Kong J., Zhang D., Zhang X. (2019). Crosstalk between Cytokinin and Ethylene Signaling Pathways Regulates Leaf Abscission in Cotton in Response to Chemical Defoliants. J. Exp. Bot..

[16] Meir S., Philosoph-Hadas S., Riov J., Tucker M.L., Patterson S.E., Roberts J.A. (2019). Re-Evaluation of the Ethylene-Dependent and -Independent Pathways in the Regulation of Floral and Organ Abscission. J. Exp. Bot..

[17] Tieman D.M., Ciardi J.A., Taylor M.G., Klee H.J. (2001). Members of the Tomato LeEIL (EIN3-like) Gene Family Are Functionally Redundant and Regulate Ethylene Responses throughout Plant Development. Plant J..

[18] Kućko A., Wilmowicz E., Pokora W., Alché J.D.D. (2020). Disruption of the Auxin Gradient in the Abscission Zone Area Evokes Asymmetrical Changes Leading to Flower Separation in Yellow Lupine. Int. J. Mol. Sci..

[19] Liang Y., Jiang C., Liu Y., Gao Y., Lu J., Aiwaili P., Fei Z., Jiang C.Z., Hong B., Ma C. (2020). Auxin Regulates Sucrose Transport to Repress Petal Abscission in Rose (Rosa Hybrida). Plant Cell.

[20] Marciniak K., Kućko A., Wilmowicz E., Świdziński M., Przedniczek K., Kopcewicz J. (2018). Gibberellic Acid Affects the Functioning of the Flower Abscission Zone in Lupinus Luteus via Cooperation with the Ethylene Precursor Independently of Abscisic Acid. J. Plant Physiol..

[21] Ma X., Li C., Huang X., Wang H., Wu H., Zhao M., Li J. (2019). Involvement of HD-ZIP I Transcription Factors LcHB2 and LcHB3 in Fruitlet Abscission by Promoting Transcription of Genes Related to the Biosynthesis of Ethylene and ABA in Litchi. Tree Physiol..

[22] Du M., Li Y., Tian X., Duan L., Zhang M., Tan W., Xu D., Li Z. (2014). The Phytotoxin Coronatine Induces Abscission-Related Gene Expression and Boll Ripening during Defoliation of Cotton. PLoS ONE.

[23] Leyser O. (2018). Auxin Signaling. Plant Physiol..

[24] Colebrook E.H., Thomas S.G., Phillips A.L., Hedden P. (2014). The Role of Gibberellin Signalling in Plant Responses to Abiotic Stress. J. Exp. Biol..

[25] Singh P., Singh A.P., Sane A.P. (2019). Differential and Reciprocal Regulation of Ethylene Pathway Genes Regulates Petal Abscission in Fragrant and Non-Fragrant Roses. Plant Sci..

[26] Werner T., Motyka V., Strnad M., Schmülling T. (2001). Regulation of Plant Growth by Cytokinin. Proc. Natl. Acad. Sci. USA.

[27] Nakano T., Fujisawa M., Shima Y., Ito Y. (2013). Expression Profiling of Tomato Pre-Abscission Pedicels Provides Insights into Abscission Zone Properties Including Competence to Respond to Abscission Signals. BMC Plant Biol..

[28] Qiu Z., Wen Z., Hou Q., Qiao G., Yang K., Hong Y., Wen X. (2021). Cross-Talk between Transcriptome, Phytohormone and HD-ZIP Gene Family Analysis Illuminates the Molecular Mechanism Underlying Fruitlet Abscission in Sweet Cherry (Prunus Avium L). BMC Plant Biol..

[29] Qi F., Zhang F. (2019). Cell Cycle Regulation in the Plant Response to Stress. Front. Plant Sci..

[30] An J.P., Yao J.F., Xu R.R., You C.X., Wang X.F., Hao Y.J. (2018). An Apple NAC Transcription Factor Enhances Salt Stress Tolerance by Modulating the Ethylene Response. Physiol. Plant.

[31] Zhao M.M., Zhang X.W., Liu Y.W., Li K., Tan Q., Zhou S., Wang G., Zhou C.J. (2020). A WRKY Transcription Factor, TaWRKY42-B, Facilitates Initiation of Leaf Senescence by Promoting Jasmonic Acid Biosynthesis. BMC Plant Biol..

[32] Huang R., Liu D., Huang M., Ma J., Li Z., Li M., Sui S. (2019). CpWRKY71, a WRKY Transcription Factor Gene of Wintersweet (Chimonanthus Praecox), Promotes Flowering and Leaf Senescence in Arabidopsis. Int. J. Mol. Sci..

[33] Zhang S., Zhao Q., Zeng D., Xu J., Zhou H., Wang F., Ma N., Li Y. (2019). RhMYB108, an R2R3-MYB Transcription Factor, Is Involved in Ethylene- and JA-Induced Petal Senescence in Rose Plants. Hortic. Res..

[34] Suttle J.C. (1988). Disruption of the Polar Auxin Transport System in Cotton Seedlings Following Treatment with the Defoliant Thidiazuron. Plant Physiol..

[35] Fu X., Shi Z., Jiang Y., Jiang L., Qi M., Xu T., Li T. (2019). A Family of Auxin Conjugate Hydrolases from Solanum Lycopersicum and Analysis of Their Roles in Flower Pedicel Abscission. BMC Plant Biol..

[36] Cin V.D., Boschetti A., Dorigoni A., Ramina A. (2007). Benzylaminopurine Application on Two Different Apple Cultivars (Malus Domestica) Displays New and Unexpected Fruitlet Abscission Features. Ann. Bot..

[37] Mishra A., Khare S., Trivedi P.K., Nath P. (2008). Effect of Ethylene, 1-MCP, ABA and IAA on Break Strength, Cellulase and Polygalacturonase Activities during Cotton Leaf Abscission. S. Afr. J. Bot..

[38] Shkolnik-Inbar D., Bar-Zvi D. (2010). ABI4 Mediates Abscisic Acid and Cytokinin Inhibition of Lateral Root Formation by Reducing Polar Auxin Transport in Arabidopsis. Plant Cell.

[39] Zhao Y., Xing L., Wang X., Hou Y.J., Gao J., Wang P., Duan C.G., Zhu X., Zhu J.K. (2014). The ABA Receptor PYL8 Promotes Lateral Root Growth by Enhancing MYB77-Dependent Transcription of Auxin-Responsive Genes. Sci. Signal.

[40] Wang X., Meng J., Deng L., Wang Y., Liu H., Yao J.L., Nieuwenhuizen N.J., Wang Z., Zeng W. (2021). Diverse Functions of IAA-Leucine Resistant PpILR1 Provide a Genic Basis for Auxin-Ethylene Crosstalk During Peach Fruit Ripening. Front. Plant Sci..

[41] Bastajian N., Friesen H., Andrews B.J. (2013). Bck2 Acts through the MADS Box Protein Mcm1 to Activate Cell-Cycle-Regulated Genes in Budding Yeast. PLoS Genet..

[42] Lyu T., Cao J. (2018). Cys2/His2 Zinc-Finger Proteins in Transcriptional Regulation of Flower Development. Int. J. Mol. Sci..

[43] Cai S., Lashbrook C.C. (2008). Stamen Abscission Zone Transcriptome Profiling Reveals New Candidates for Abscission Control: Enhanced Retention of Floral Organs in Transgenic Plants Overexpressing Arabidopsis ZINC FINGER PROTEIN2. Plant Physiol..

[44] Gan Y., Liu C., Yu H., Broun P. (2007). Integration of Cytokinin and Gibberellin Signalling by Arabidopsis Transcription Factors GIS, ZFP8 and GIS2 in the Regulation of Epidermal Cell Fate. Development.

[45] Zhou Z., Sun L., Zhao Y., An L., Yan A., Meng X., Gan Y. (2013). Zinc Finger Protein 6 (ZFP6) Regulates Trichome Initiation by Integrating Gibberellin and Cytokinin Signaling in Arabidopsis Thaliana. New Phytol..

[46] Guo P., Li Z., Huang P., Li B., Fang S., Chu J., Guo H. (2017). A Tripartite Amplification Loop Involving the Transcription Factor WRKY75, Salicylic Acid, and Reactive Oxygen Species Accelerates Leaf Senescence. Plant Cell.

[47] Gu L., Ma Q., Zhang C., Wang C., Wei H., Wang H., Yu S. (2019). The Cotton GhWRKY91 Transcription Factor Mediates Leaf Senescence and Responses to Drought Stress in Transgenic Arabidopsis Thaliana. Front. Plant Sci..

[48] Zhang D., Zhu Z., Gao J., Zhou X., Zhu S., Wang X., Wang X., Ren G., Kuai B. (2021). The NPR1-WRKY46-WRKY6 Signaling Cascade Mediates Probenazole/Salicylic Acid-Elicited Leaf Senescence in Arabidopsis Thaliana. J. Integr. Plant Biol..

[49] Liao W., Yang Y., Li Y., Wang G., Peng M. (2016). Genome-Wide Identification of Cassava *R2R3 MYB* Family Genes Related to Abscission Zone Separation after Environmental-Stress-Induced Abscission. Sci. Rep..

[50] Livak K.J., Schmittgen T.D. (2001). Analysis of Relative Gene Expression Data Using Real-Time Quantitative PCR and the 2−ΔΔCT Method. Methods.

[51] Yang J., Zhang J., Wang Z., Zhu Q., Wang W. (2001). Hormonal Changes in the Grains of Rice Subjected to Water Stress during Grain Filling. Plant Physiol..

[52] Zhao J., Li G., Yi G.X., Wang B.M., Deng A.X., Nan T.G., Li Z.H., Li Q.X. (2006). Comparison between Conventional Indirect Competitive Enzyme-Linked Immunosorbent Assay (IcELISA) and Simplified IcELISA for Small Molecules. Anal. Chim. Acta.

[53] Xue J., Li Y., Tan H., Yang F., Ma N., Gao J. (2008). Expression of Ethylene Biosynthetic and Receptor Genes in Rose Floral Tissues during Ethylene-Enhanced Flower Opening. J. Exp. Bot..

