# Supplementary figures and images for: Thidiazuron Promotes Leaf Abscission by Regulating the Crosstalk Complexities between Ethylene, Auxin, and Cytokinin in Cotton
Source: Int J Mol Sci. 2022 Feb 28;23(5):2696. doi: 10.3390/ijms23052696 (PMC8910847; doi:10.3390/ijms23052696)

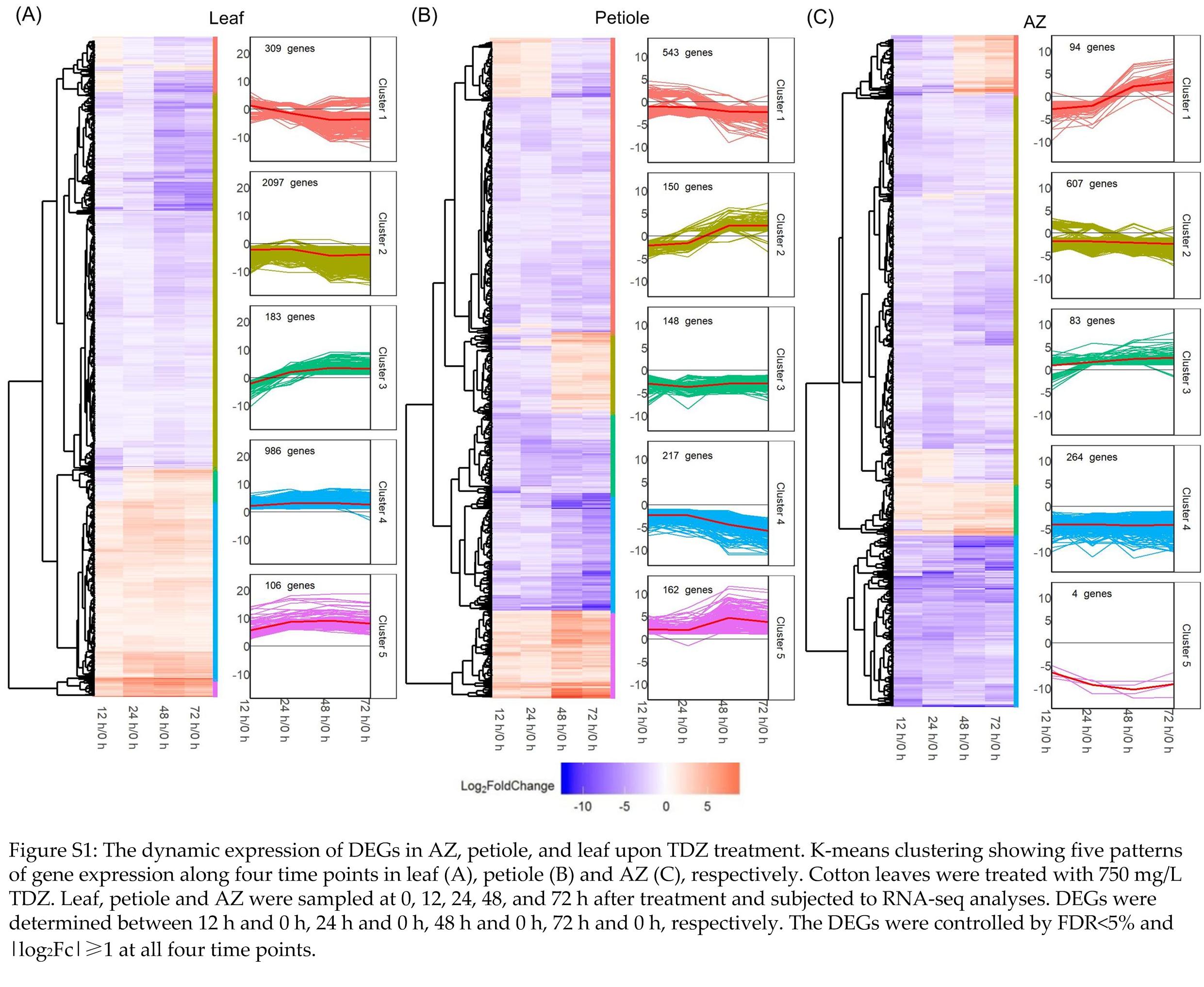

Supplement: Supplementary file 1 [file ijms-23-02696-s001.zip › FIGS1.png]

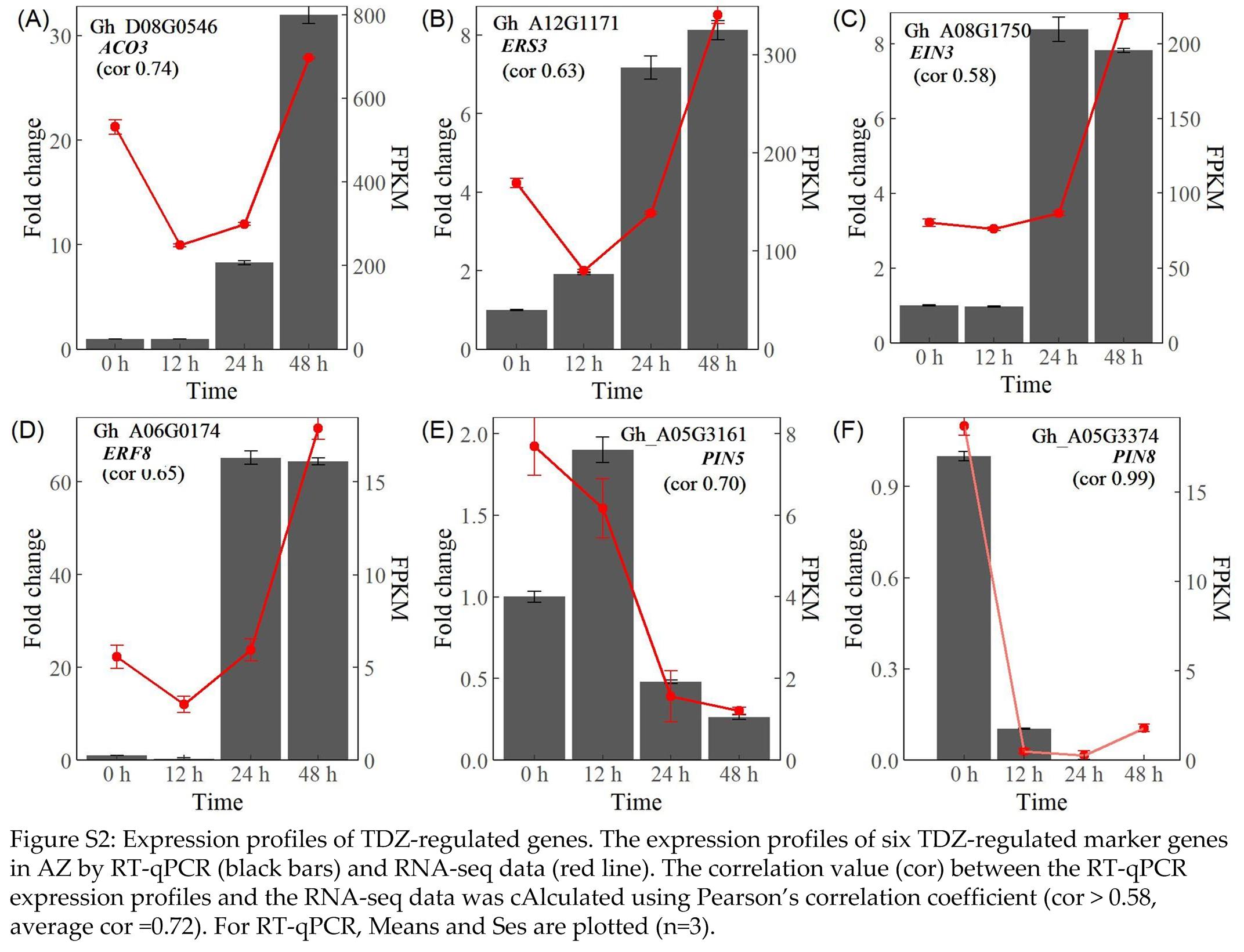

Supplement: Supplementary file 1 [file ijms-23-02696-s001.zip › FIGS2.png]

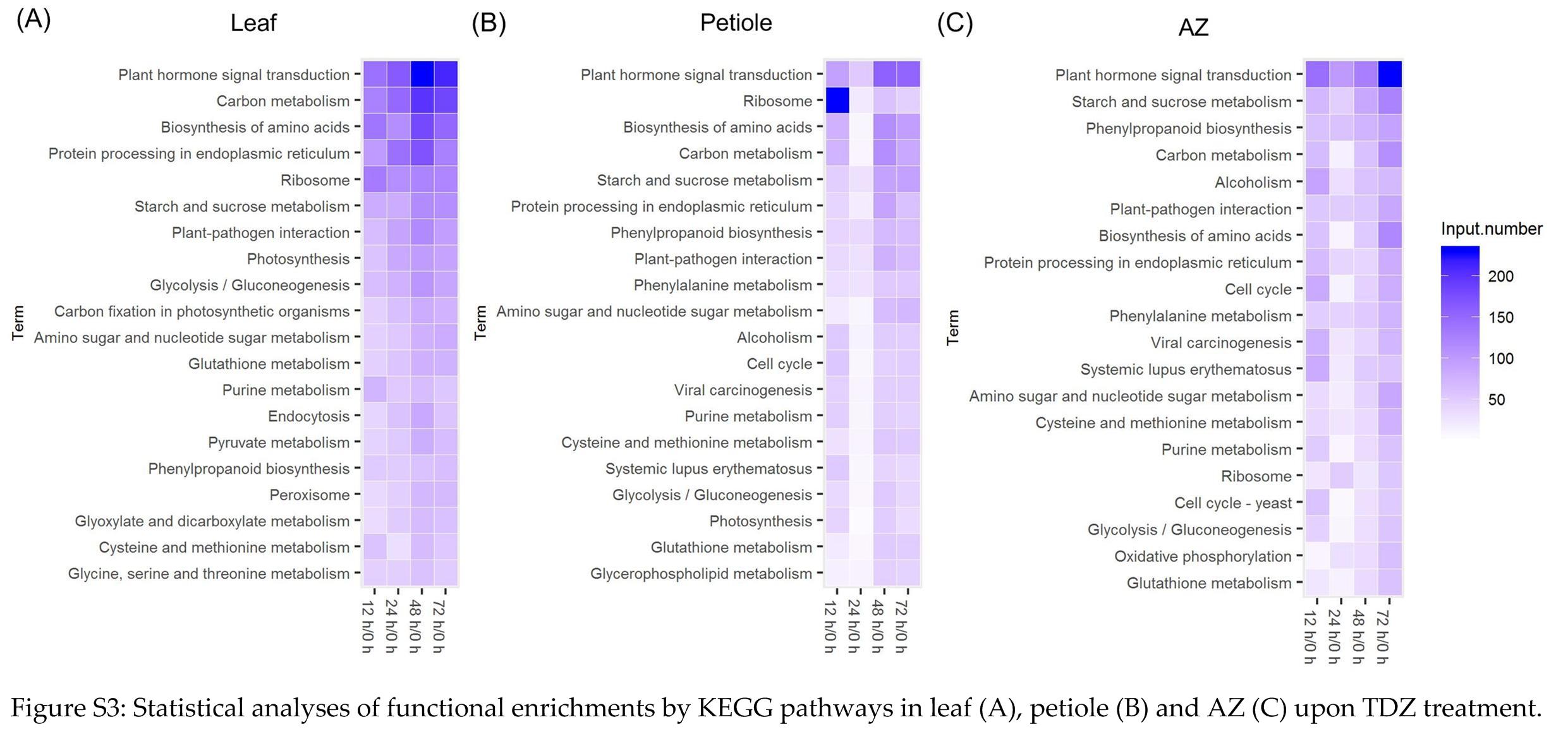

Supplement: Supplementary file 1 [file ijms-23-02696-s001.zip › FIGS3.png]

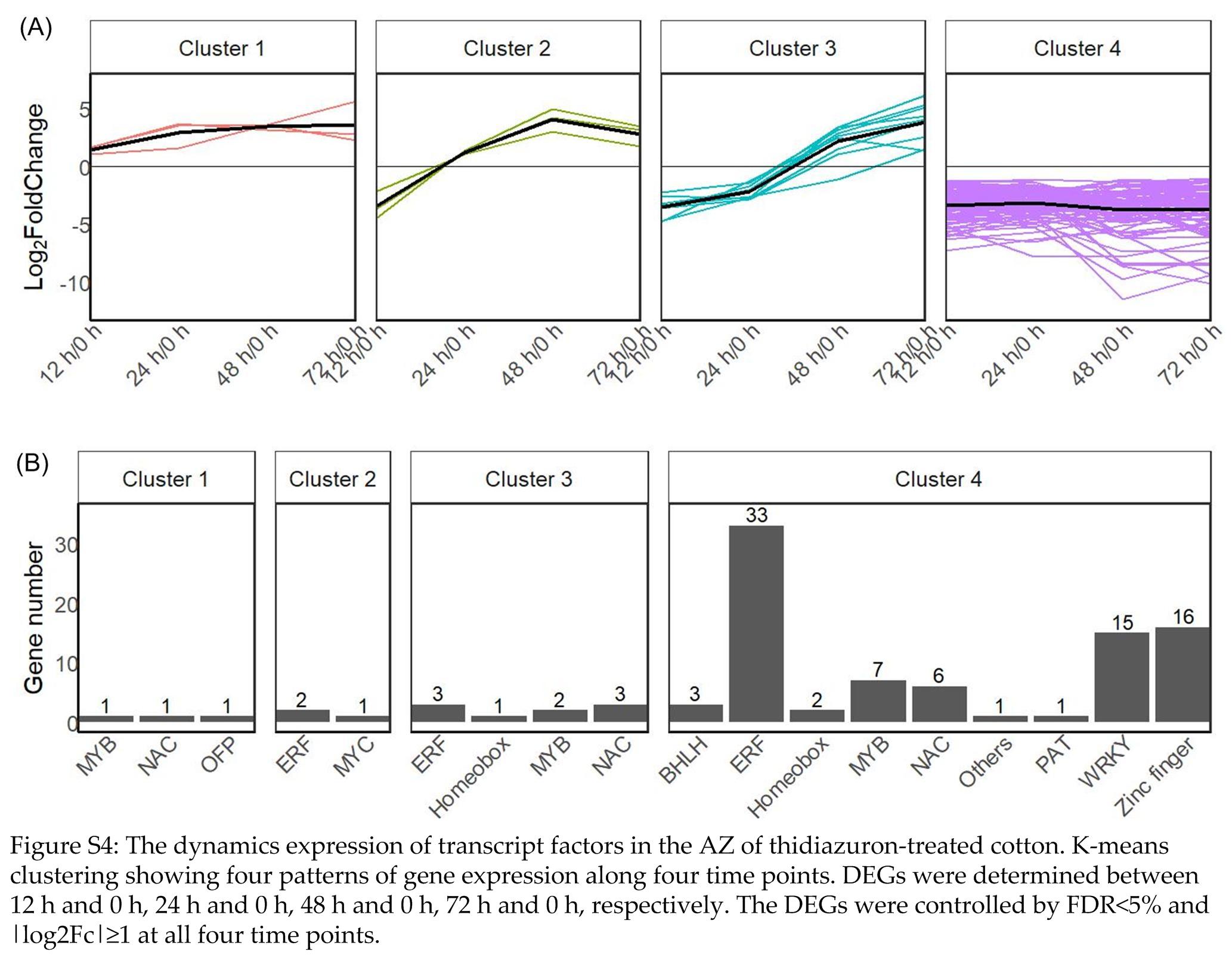

Supplement: Supplementary file 1 [file ijms-23-02696-s001.zip › FIGS4.png]
